# Estimation Method of Soluble Solid Content in Peach Based on Deep Features of Hyperspectral Imagery

**DOI:** 10.3390/s20185021

**Published:** 2020-09-04

**Authors:** Baohua Yang, Yuan Gao, Qian Yan, Lin Qi, Yue Zhu, Bing Wang

**Affiliations:** 1 School of Information and Computer, Anhui Agricultural University, Hefei 230036, China; ybh@ahau.edu.cn (B.Y.); gaoyuan@ahau.edu.cn (Y.G.); qiqilin@ahau.edu.cn (L.Q.); zhuyue@ahau.edu.cn (Y.Z.); 2 School of Electrical and Information Engineering, Anhui University of Technology, Ma’anshan 243032, China; yanqian201288@163.com

**Keywords:** soluble solids content, hyperspectral imagery, random forest, peach, deep features

## Abstract

Soluble solids content (SSC) is one of the important components for evaluating fruit quality. The rapid development of hyperspectral imagery provides an efficient method for non-destructive detection of SSC. Previous studies have shown that the internal quality evaluation of fruits based on spectral information features achieves better results. However, the lack of comprehensive features limits the accurate estimation of fruit quality. Therefore, the deep learning theory is applied to the estimation of the soluble solid content of peaches, a method for estimating the SSC of fresh peaches based on the deep features of the hyperspectral image fusion information is proposed, and the estimation models of different neural network structures are designed based on the stack autoencoder–random forest (SAE-RF). The results show that the accuracy of the model based on the deep features of the fusion information of hyperspectral imagery is higher than that of the model based on spectral features or image features alone. In addition, the SAE-RF model based on the 1237-650-310-130 network structure has the best prediction effect (R^2^ = 0.9184, RMSE = 0.6693). Our research shows that the proposed method can improve the estimation accuracy of the soluble solid content of fresh peaches, which provides a theoretical basis for the non-destructive detection of other components of fresh peaches.

## 1. Introduction

Peach is a kind of fruit loved by different consumers because of its high nutrition and unique taste and flavor. As an important component of peaches, soluble solids content (SSC) is an important index to measure the flavor of peaches [1]. SSC is also the basic raw material for synthetic vitamins and carotenoids and other nutrients, which plays an important role in guiding peach harvest time and post-harvest storage and processing [2]. However, the traditional destructive detection methods can no longer meet the high precision requirements [3]. Therefore, the effective detection of SSC content has important research significance and application value.

In recent years, with the rapid development of sensors, non-destructive detection technology has been used in fruit quality evaluation. For example, multispectral [4,5], fluorophore [6,7,8], near-infrared spectroscopy [9,10,11,12], electronic nose [13,14,15], and dielectric technology [16,17] have been applied for the evaluation of the soluble solid content in fresh fruits. Among these technologies, near-infrared spectroscopy is currently the most widely used method for evaluating fresh fruit SSC due to its fast, simple, and non-destructive characteristics. However, lacking the spatial characteristics of fruit hyperspectral images limits the further exploration of fruit SSC prediction models.

At present, hyperspectral imaging (HSI) is widely applied for the detection of soluble solids in fruits due to more comprehensive information [18,19,20,21,22,23,24,25]. However, most of the studies mentioned above are only based on the analysis of spectral information, which ignores the image information of the detected object in the hyperspectral image. Although Fan et al. proposed an SSC detection method that combines spectral and texture features [26], the robustness of the constructed prediction model still needs to be further improved. Li et al. estimated the SSC content of fresh peaches based on hyperspectral images [27]. However, the analysis of sensitive spectral information alone cannot provide the deep features of HIS, which are robust features extracted from deep neural networks, resulting in low accuracy of the model. Therefore, non-destructive detection based on SSC is facing great challenges.

In fact, the amount of hyperspectral image data is huge and the correlation between the bands is strong, which lead to the redundancy in HSI. If the hyperspectral image is directly analyzed, the robustness of the constructed model will be poor. Therefore, how to effectively fuse various features of hyperspectral images faces many problems: on the one hand how to extract more comprehensive features and on the other hand how to determine more typical features. Previous studies have shown that methods based on successive projections algorithm (SPA) [28], principal component analysis (PCA) [29], and random forest (RF) [30] have achieved good results for the feature selection and dimensionality reduction from hyperspectral images. However, there are still some obstacles to the hyperspectral image feature acquisition of fresh peaches.

The wide application of deep learning in different fields provides new ideas for the prediction of soluble solid content of fresh peaches. As an unsupervised deep learning technology, stacked autoencoder (SAE) has the capabilities of deep feature extraction and dimensionality reduction processing, thereby providing comprehensive and typical features for prediction models [31,32]. Many studies have shown that the spectral features extracted based on the SAE method can improve the accuracy of the estimation model for fruit SSC [33,34]. Although the above-mentioned studies have successfully detected the soluble solid content of different fruits, it is still unclear whether the performance of the estimation model of peach soluble solid content can be improved by fusing features.

Therefore, the deep features are extracted separately based on SAE from the spectral information (reflectance) and image information (pixels) and fusion information of hyperspectral images, aiming to improve the estimation accuracy of peach soluble solids. The purpose of this study was: (1) to obtain key information from HSI on the internal and external quality of fresh peaches at different levels of ripeness; (2) to extract the deep features of spectral information and image information based on SAE to achieve a better representation of HSI features; and (3) to construct a random forest prediction model with deep features to quickly detect the SSC content of fresh peaches.

## 2. Materials and Methods

### 2.1. Sample Collection

From April to June 2019, 120 peach samples named “Golden Peach” were collected at the Orchard Base in Hefei, Anhui Province, China, including three different maturities (30 immature, 50 semi-mature, and 40 mature peaches). After the surface of all peaches were cleaned, they were stored in an environment of 25 °C for 12 h, so that the sample temperature was basically the same as room temperature.

### 2.2. Data Collection

#### 2.2.1. Hyperspectral Image Acquisition

**Figure 1:**
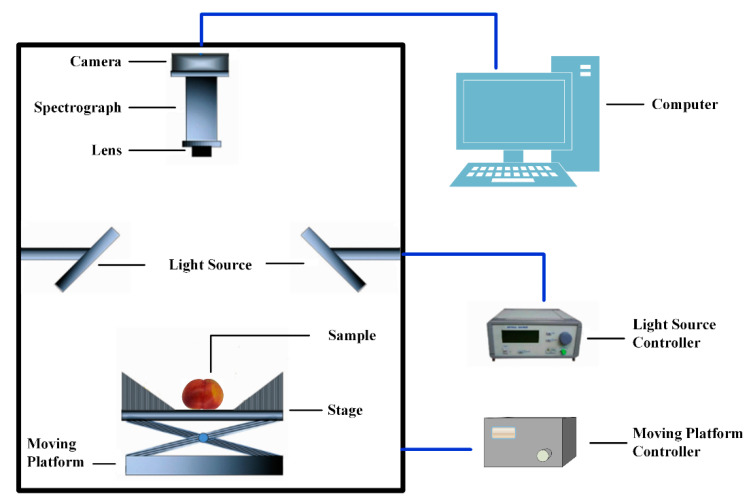
Hyperspectral imaging system.

The hyperspectral image acquisition system used in this research includes a spectral imager (Imspector V17E, Spectral Imaging Ltd., Oulu, Finland), a camera as a CCD camera (IPX-2M30, Imperx Inc., Boca Raton, FL, USA), two 150-W halogen lamps (3900, Illumination Technologies Inc., New York, NY, USA), a data acquisition dark box, image acquisition and analysis software (Spectral Image Software, Isuzu Optics Corp., Taiwan, China), reflective linear optical path Tube, and electronically controlled displacement platform (MTS120, Beijing Optical Instrument Factory, Beijing, China). The illumination direction of the light source was 45° from the vertical direction, and the entire collection system was placed in a dark box, as shown in Figure 1.

To obtain high-quality images, the distance from the highest point of the peach sample to the objective lens was 220 mm, and the motor control speed and exposure time were set to 0.8 mm/s and 2 ms, respectively. The spectral resolution of the system was 5 nm, and the image resolution was set to 636 pixels × 838 pixels. To reduce the influence of image noise and dark current as much as possible, after scanning the peach sample, the standard white and dark reference images were used to calibrate the obtained hyperspectral data [35].

#### 2.2.2. Peach Soluble Solids Content Collection

The SSC of the peach sample was measured by a handheld refractometer (Model: LYT-330, Shanghai Linyu Trading Co., Ltd., Shanghai, China) with temperature automatic compensation function, which has a measurement range of 0–32 °Brix and a resolution of 0.2 °Brix. When the SSC of the peach sample was measured, the pulp about 6-mm deep in the sample spectrum collection part was selected for squeezing, which was dropped on the detection window on the brix meter, and then the data were recorded. The average value of each sample repeated 3 times was the final SSC value of the sample. The statistical results of the corrected set and the predicted set of the measured peach sample SSC are shown in Table 1.

### 2.3. Methodology

#### 2.3.1. Stacked Autoencoder

Autoencoder (AE) is mainly composed of encoder and decoder. The model can learn the most important attributes of the input data and reconstruct the input data in the output through encoding and decoding [36]. Generally, the stage of mapping the input data to the hidden layer through a nonlinear activation function is called encoding, and the mapping of the hidden layer to the output layer is called decoding. Therefore, to some extent, AE is a small deep learning model, which mainly includes input layer, hidden layer, and output layer.

Take a dataset {$x_{1},x_{2}\ldots x_{m}$}, where m is the number of training samples. In the coding stage, the training samples can be encoded to obtain the feature expression of the hidden layer, as shown in Equation (1). $y_{i}$ is the activation value of the neural unit of the hidden layer. In the decoding stage, the network reconstructs the data to obtain the output closest to the original data, as shown in Equation (2). $z_{i}$ is the inverse conversion of the activation value into the reconstruction of the input sample. Equation (3) represents the loss function of SAE. J(x,z) is the value of the error function [37,38].

(1) $$y_{i}=g(wx_{i}+b_{1})$$

(2) $$z_{i}=g(w^{T}y_{i}+b_{2})$$

(3) $$J(x,z)=\frac{1}{2m}{\sum_{i=1}^{m}\left\|z_{i}-x_{i}\right\|}^{2}$$

where i=1,2,3…m, w is the weight matrix connecting the input layer and the hidden layer, $w^{T}$ represents the weight matrix connecting the hidden layer and the output layer, $b_{1}$ represents the offset between the input layer and the hidden layer, $b_{2}$ represents the offset between the hidden layer and the output layer, g(x) is the activation function, and Sigmoid and ReLU are commonly activation functions.

Stacked autoencoder (SAE) are superimposed on multiple autoencoders, and each autoencoder is trained individually in an unsupervised way using a greedy layer-by-layer training method, which can solve the gradient of traditional multi-layer neural network training problem [39]. The output of the previous hidden layer is used as the input of the next hidden layer. After the model is pre-trained, the refinement adjustment is performed to obtain the optimal parameters, and the output of the last hidden layer is used as the deep feature of the input data.

#### 2.3.2. Information Extraction from Hyperspectral Images

To obtain spectral features and image features, it is necessary to extract the original information of the spectrum and the image. First, 636 × 838 × 508 hyperspectral images were obtained from each peach sample through HSI. Second, to obtain the spectral information, 200 × 200 pixels graphic regions of interest (ROIs) were selected at the vicinity of the equator of the peach, where the average spectral reflectance of all pixels was extracted as the spectral data of the sample. Then, to obtain the image information, the images of each band in the ROI were extracted, saved as 457 RGB images (200 × 200 pixels) of peaches, and converted to grayscale images, with the size unified to 28 × 28 pixels. Finally, to obtain more comprehensive information, the original information of the spectral reflectance and image pixels was fused.

#### 2.3.3. Stacked Autoencoder–Random Forest

**Figure 2:**
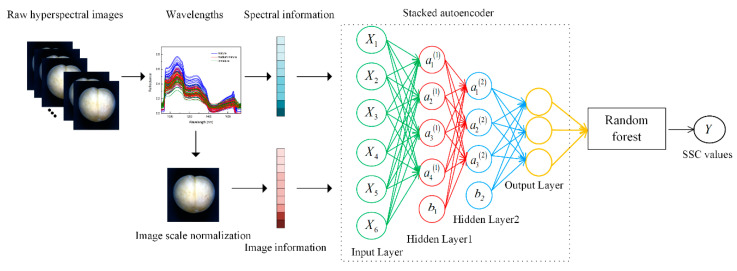
Structure of the stacked autoencoder–random forest (SAE–RF).

To predict the SSC of fresh peaches, a stacked autoencoder–random forest (SAE-RF) model was proposed in this study. Based on the deep features of the spectrum, the deep features of the image and the deep features of the fusion information extracted by SAE, a random forest algorithm (RF) was used to establish a prediction model for the soluble solid content of peaches. Figure 2 shows the SAE-RF model based on fusion information. SAE includes two hidden layers, and Features 1 and 2 are deep features extracted from the fusion information based on the hidden layer 1 and hidden layer 2. Feature 2 was used as the input of the random forest algorithm to predict the soluble solid content of peaches. Finally, the coefficient of determination (R^2^) and root mean squared error (RMSE) [40] were used as evaluation indicators to explain and quantify the relationship between the soluble solid content of peaches and deep features.

## 3. Results and Analysis

### 3.1. Deep Feature Extraction Results of Hyperspectral Images

**Figure 3:**
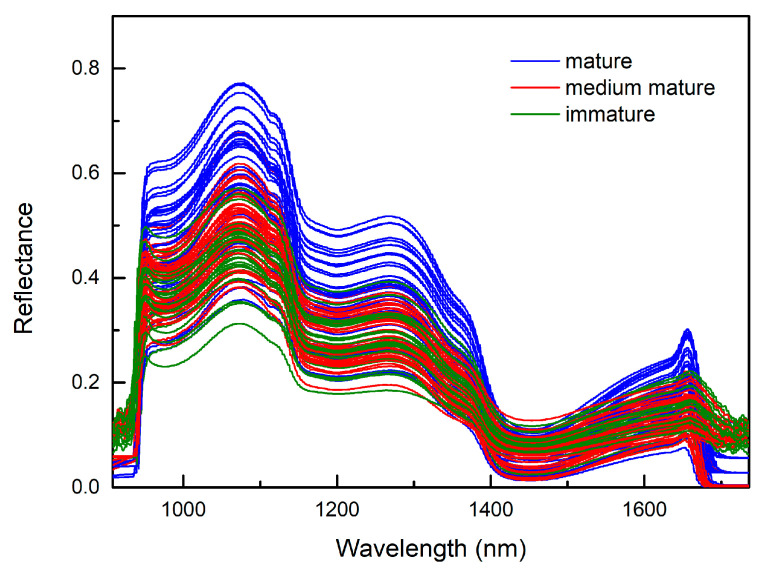
Hyperspectral original curve of fresh peach.

In total, 508 wavelength bands (908.1–1735.6 nm) were obtained from the peach samples, as shown in Figure 3. To improve the stability of the model, it was necessary to delete some of the bands at the beginning and the end with obvious noise, including 21 bands of 908–940 nm and 34 bands of 1681–1735 nm. The remaining 453 bands within 942–1680 nm were used as spectral data. The hyperspectral curve of fresh peach samples can effectively represent the chemical information of the main components such as SSC in fresh peaches, and there is a certain correlation between the spectral reflection intensity and the SSC content. Therefore, the spectral reflectance intensities of samples with different SSCs at different wavelength bands are quite different. In the range of 1000–1300 nm, the spectral reflectance of different samples varies greatly. In the range of 1400–1520 nm, the difference in spectral reflectance of different samples is small. In addition, the spectral curve changes due to the combined frequency of the H group vibration and the absorption of multiple frequency doublings in the soluble solid content of peach.

To obtain more accurate image information, the three peaks of the spectral curve, 1070, 1270, and 1650 nm, were selected as the center wavelengths, and images in the three bands were acquired, which include 1050–1090, 1250–1290, and 1630–1670 nm. The gray-scale mean value corresponding to each image pixel was extracted separately and converted into a one-dimensional vector, which contains 784 pixels, and the data dimension corresponding to each image is 784. To improve the computational efficiency, the image vector was normalized and used as the input of SAE, and the output was the deep features of image.

**Figure 4:**
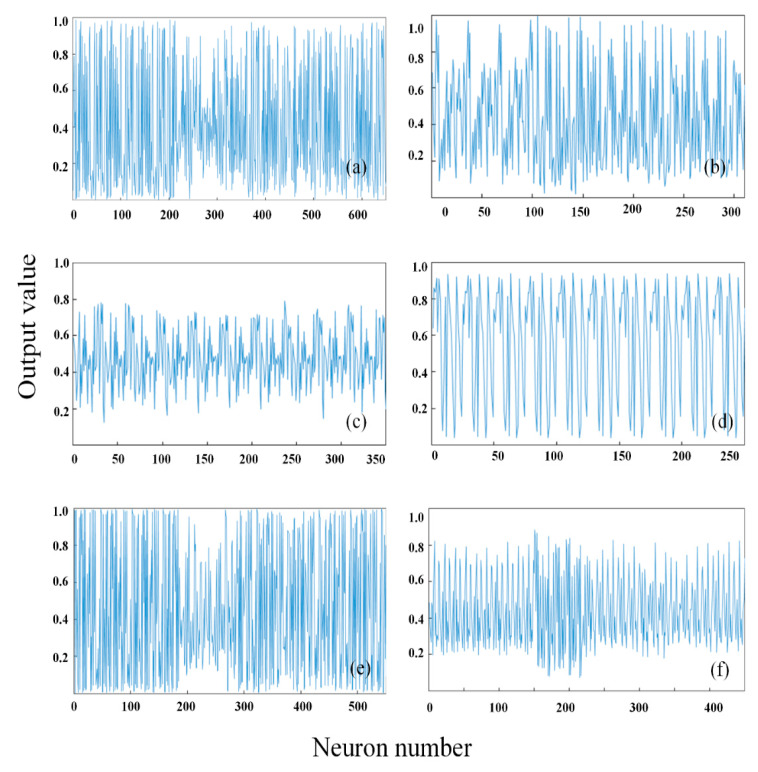
Output data of different hidden layers of stacked autoencoder: (Left) the output data of the first layer; and (Right) the output data of the second layer of spectral. (**a**,**b**) SAE structure 453-350-200-60; (**c**,**d**) SAE structure 784-550-450-70 and the fusion information; and (**e**,**f**) SAE structure 1237-650-310-130.

To obtain more comprehensive hyperspectral image features, the spectral reflectance with 453 dimensions and the image pixels with 784 dimensions were fused to obtain fusion information with 1273 dimensions. The fusion vectors were normalized as the input of SAE, and the output were the deep features of the fusion information. Figure 4a,b shows the output results of the deep features based on the spectral information of the two hidden layers of SAE (the first hidden layer had 350 neurons, the second hidden layer had 200 neurons, and the output was 60 features). Figure 4c,d shows the output result of the deep features based on the image information of the two hidden layers of SAE (the first hidden layer had 550 neurons, the second hidden layer had 450 neurons, and the output was 130 features). Figure 4e,f shows the output result of the deep features based on the fusion information of the two hidden layers of SAE (the first hidden layer had 650 neurons, the second hidden layer had 310 neurons, and the output was 130 features).

### 3.2. Different Structures of SAE for Peach to Estimate Soluble Solids Content

Spectral data (453 dimensions), image pixels (784 dimensions), and fusion information (1237 dimensions) were separately used as input data of SAE to extract deep features, which were used as the input to the random forest algorithm to construct a prediction model for the soluble solid content of peaches. The results are shown in Table 2. Table 2 shows that, regardless of the number of neurons in the hidden layer of SAE, the prediction effect of the model based on the deep features extracted from the fusion information is the best. The model prediction of the fusion information reduced from 1237 to 130 dimensions has the best effect among the three structures (1237-750-300-40, 1237-600-460-90, and 1237-650-310-130). For calibration set, the accuracy of the 130-dimensional model is 2.9% higher than that of the model based on 40-dimensional deep features and 1.9% higher than that of the 90-dimensional model. Corresponding to the validation set, the 130-dimensional model is improved by 3.1% and 1.1%, respectively. The result shows that the data dimension and the accuracy of the model are not positively correlated.

### 3.3. Comparison of Estimation Models of Peach SSC Based on Different Features

**Figure 5:**
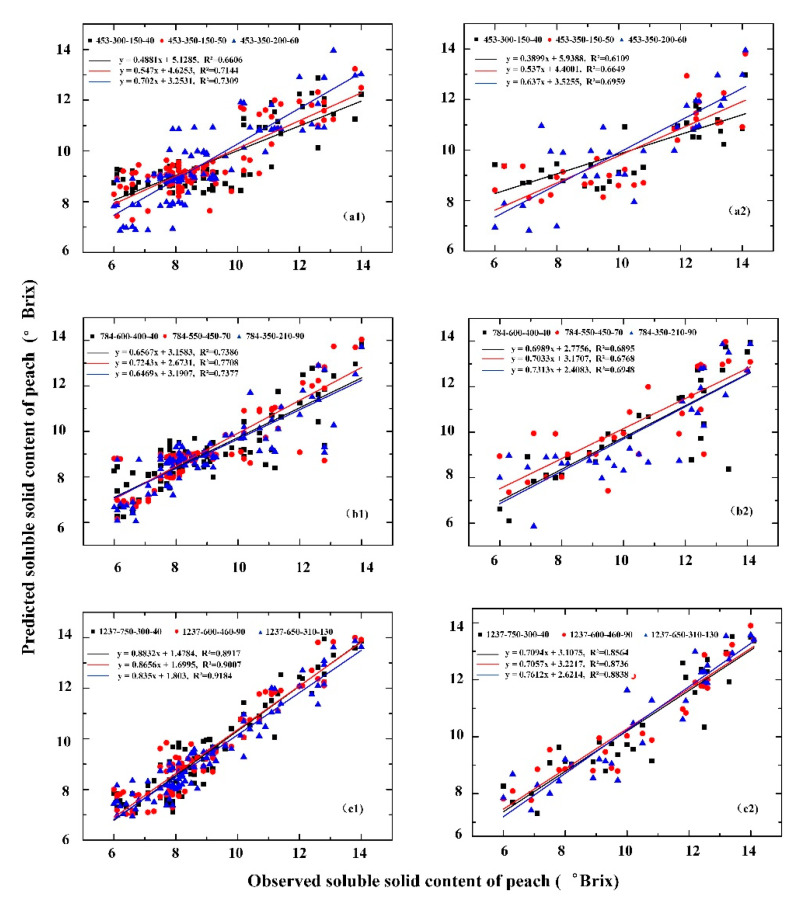
Comparison of the prediction results of the SAE–RF models based on different features: (Left) calibration set; and (Right) validation set. The deep features of: spectrum (**a1**,**a2**); image (**b1**,**b2**); and fusion information (**c1**,**c2**).

From the comparison of the models based on different features shown in Figure 5, we can easily find that the prediction effects of models based on different deep features have certain differences. For calibration set, the model R^2^ based on the deep features of the spectrum is 0.6606–0.7309, and the SAE based on the structure of 453-350-200-60 has the best prediction effect of the three different structures (453-300-150-40, 453-350-150-50, and 453-350-200-60). The R^2^ of model based on the deep features of the image is 0.7377–0.7708, and the prediction effect based on the SAE structure of 784-550-450-70 is best in the three structures (784-600-400-40, 784-550-450-70, and 784-350-210-90). The model R^2^ based on the deep features of the fusion information is 0.8917–0.9184, and the prediction effect based on the SAE structure of 1237-650-310-130 is the best in the three structures (1237-750-300-40, 1237-600-460-90, and 1237-650-310-130). For validation set, the R^2^ of model based on the deep features of spectrum, deep features of image, and deep features of fusion information are 0.6109–0.6959, 0.6747–0.6948, and 0.8564–0.8838, respectively.

## 4. Discussion

### 4.1. Parameter Selection and Experimental Results

The experimental environment configuration for deep learning in this research was as follows. The motherboard was Z370 HD3-CF, the CPU was Intel Core i7-8700, the video memory was 8 GB GDDR5, and the memory was 16 GB. The software experiment environment configuration was: the operating system was Windows 10 64-bit; the programming software and language were Anaconda 3, MATLAB 2017 and Python 3.6, respectively; and the deep learning framework was Keras.

The number of neurons in the hidden layer directly affects the feature extraction capability of SAE, which plays a key role in the accuracy of the model. To ensure that effective features are found from the training set and achieve the expected prediction accuracy, it is necessary to find a balance between the SAE feature extraction of unsupervised training and the fine-tuning of supervised training [41]. On the one hand, if there are too few neurons, the model is prone to overfitting. On the other hand, too many neurons will lead to longer training time. Therefore, the SAE model was trained according to different input data, and different numbers of hidden layer neurons were selected.

For spectral information, 350 neurons were selected in the first layer, and 200 and 150 neurons were selected in the second layer. It was found that the model effect of 200 neurons is better than that of 150 neurons. For image information, the first layer was 600, 550, and 350 neurons, and the second layer was 400, 450, and 210 neurons. It was found that the models with 550 and 450 neurons perform best. For fusion information, the first layer was 750, 650, and 600 neurons, and the second layer was 300, 460, and 310 neurons. It was found that the models with 650 and 310 neurons perform best.

### 4.2. Different Structures and Different Features

The accuracy of the model based on deep features of fusion information with a structure of 1237-650-310-130 is the highest regardless of the calibration set or the validation set, which are 20.4% and 21.2% higher than those of the model based on deep features of spectrum (SAE structure is 453-350-200-60), and 16.1% and 23.6% higher than those of the model based on deep features of image (SAE structure is 784-550-450-70). Research shows that the deep features of fusion of spectral information and image pixel information can more comprehensively reflect the external and internal quality of fresh peach samples, and effectively improve the performance of the model for predicting SSC in peach.

Traditional hyperspectral image processing methods mainly extract features such as spectrum, color, and texture [42]. However, models based on these features often have certain limitations. In this study, the hyperspectral image was first separated by band, and the average gray value of the image was obtained as the characteristic image of the sample. In addition, the typical spectrum data were extracted as the feature wavelength. SAE was used to extract deep features, combined with random forest algorithm to establish a model to predict the SSC in peaches, which not only improves its feature expression ability, but also effectively improves the robustness of the model. Although SAE models with different structures were designed, the proposed model achieves good prediction performance. In the future, it is necessary to study comparative experiments based on deep learning models with different frameworks to provide new technical support for SSC prediction of different fruits.

### 4.3. Visualized Results of Soluble Solid Content

**Figure 6:**
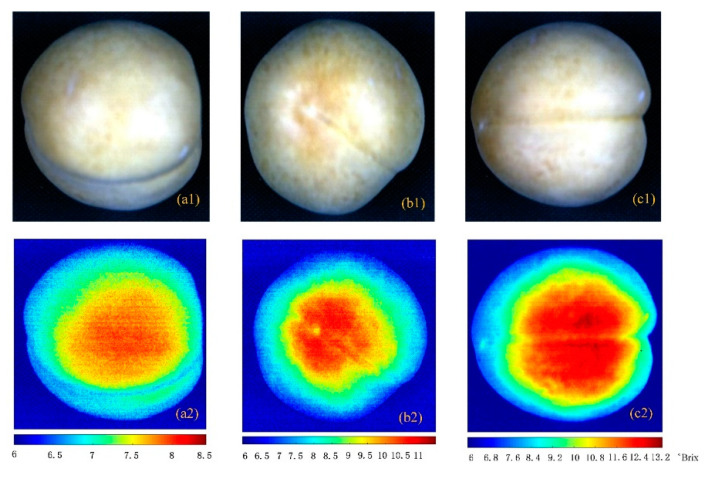
Visualization of peach SSC with different maturity: (**a1**–**c1**) the hyperspectral images of immature, medium ripe, and mature peach; and (**a2**–**c2**) the pseudo-color images of immature, medium mature, and mature peach.

The visualization results of the soluble solid content in peaches are shown in Figure 6. Peach samples of different maturity levels were used as test samples. In Figure 6, the first row represents the original hyperspectral images of fresh peaches and the second row represents the corresponding pseudo-color image. The soluble solid content indicator bar below the graph shows the range of soluble solid content in fresh peaches of different maturity. The soluble solid content of immature peaches, medium ripeness, and mature peaches range 0–8.5, 0–11, and 0–13.2 °Brix, respectively. The figure shows the soluble solids and distribution of fresh peaches of different maturity. It is easy to find that the soluble solids are mainly distributed in the center of the peaches, and the soluble solids of the immature peaches are lower than those of the medium mature ones, that is, the soluble solids of the fresh peaches gradually increase as they mature.

## 5. Conclusions

Soluble solids content (SSC) is a key component to evaluate the quality of fresh peaches. Hyperspectral images provide an efficient method for non-destructive detection of peach SSC. In this paper, a model for estimating the soluble solid content of peaches based on the deep features of the fusion information which include spectrum and image is proposed. Deep learning theory is applied to the estimation of the soluble solid content of peaches, and the SAE feature extraction of unsupervised training is combined with the fine-tuning of supervised training. On the one hand, compared with spectral and image deep feature modeling, the deep feature model based on the fusion information is more effective, which shows that the fusion information contains more comprehensive sample features. On the other hand, SAE-RF models based on different network structures were constructed to estimate the soluble solid content in peaches. Among them, the model based on fusion features (network structure is 1237-650-310-130) has the highest accuracy (R^2^ = 0.9184 for calibration, R^2^ = 0.8838 for validation), which provides a new method for quantitatively estimating the soluble solid content of peaches and realizes the SSC monitoring of peaches based on internal and external quality. In addition, the detection of peach quality parameters based on different deep learning methods, such as the detection of acidity, hardness, and moisture in peach, which will be further carried out in the future.

## Figures and Tables

**Table 1:** Soluble solids content (SSC) distributions of peaches.

Dataset	Number	Content Range (%)	Mean (%)	SD (%)
Full	120	6–14.1	9.29	2.17
Calibration set	90	6–14	8.91	1.94
Validation	30	6–14.1	10.41	2.41

SD: standard deviation.

**Table 2:** Evaluation model of peach SSC based on different SAE structures and different features.

Features	SAE Optimal Scale	Calibration Set		Validation Set	
		R^2^	RMSE	R^2^	RMSE
Deep feature of spectral	453-300-150-40	0.6606	1.3323	0.6109	1.699
	453-350-150-50	0.7144	1.2551	0.6649	1.5033
	453-350-200-60	0.7309	1.1744	0.6959	0.2486
Deep feature of image	784-600-400-40	0.7386	1.0163	0.6895	1.3886
	784-550-450-70	0.7708	0.9613	0.6747	1.3733
	784-350-210-90	0.7377	1.018	0.6948	1.3825
Deep feature of fusion information	1237-750-300-40	0.8917	0.7756	0.8564	0.9922
	1237-600-460-90	0.9007	0.7956	0.8736	0.9715
	1237-650-310-130	0.9184	0.6693	0.8838	0.8887

Note: The optimal scale represents the SAE data dimension and the optimal number of neurons in each layer.
